# Legionella stuttgartensis sp. nov. and Legionella nigrisilvae sp. nov., two new species isolated from a re-cooling plant and from a drinking water house installation in southern Germany

**DOI:** 10.1099/ijsem.0.007187

**Published:** 2026-06-02

**Authors:** Bibiana Rios Galicia, Patryk Krauze, Caroline Hengerer, Konstantin Licht, Stefan Brockmann, Jens Fleischer

**Affiliations:** 1 Department of Health Protection, Infection Control and Epidemiology, Baden-Wuerttemberg Federal State Health Office, Ministry of Social Affairs, Health and Integration, Stuttgart, Germany

**Keywords:** drinking water, drinking water house installation, *Legionella nigrisilvae* sp. nov., *Legionella stuttgartensis* sp. nov., new species, re-cooling plant, whole-genome sequencing (WGS)

## Abstract

The genus *Legionella* encompasses environmental bacteria frequently present in water samples. Two strains of *Legionella* isolated from a re-cooling plant and from a drinking water house installation in southern Germany were identified as two different novel species. The isolates were both Gram-stain negative rods, negative to latex agglutination, able to grow at 37 °C on buffered charcoal–yeast extract agar with l-cysteine agar. The phenotypic characterization was complemented by whole-genome sequencing. The genomes of the strains WA2022007384 and WA2024007413 were sequenced and identified as two different novel species with a GC mol% content of 37.92 and 37.91, respectively. Both strains encoded antimicrobial resistance genes against beta-lactams, aminoglycosides and clusters for legioliulin biosynthesis that might confer fluorescence under UV light at 365 nm. The average nucleotide identity percentage between the strain WA2022007384 and its closest related strains was 87.41% to *Legionella qingyii* km489^T^ and 86.97% to *Legionella gormanii* NCTC11401^T^. For the strain WA2024007413, the closest species were 98.28% to *Legionella* sp. PC997, 85.84% to *L. gormanii* NCTC11401^T^ and 85.61% to *L. qingyii* km489^T^. The phylogenetic tree based on 164 concatenated core genes showed a separated clade neighbouring the strains *L. gormanii* NCTC11401^T^ and *L. qingyii* km489^T^. These results confirm the description of the two novel species *Legionella stuttgartensis* sp. nov., with WA2022007384^T^ (=DSM 120481^T^=NCCB 101094^T^) as its type strain, and *Legionella nigrisilvae* sp. nov., with WA2024007413^T^ (=DSM 120480^T^=NCCB 101095^T^) as its type strain.

## Introduction

Species from the genus *Legionella* are Gram-negative aerobic rods found in sweet-water environments, water installations or in single-celled protozoa as natural hosts and reservoirs [1]. Some species are responsible for sporadic and epidemic outbreaks of atypical pneumonia, including the well-known legionellosis, Legionnaire’s disease caused mainly by *Legionella pneumophila* serogroup 1 (Lp1) [2,3].

The List of Prokaryotic names with Standing in Nomenclature (LPSN database accessed on 9 September 2025) recognizes 69 species of *Legionella*, 1 of which is described only as a metagenome assembly: Candidatus *Legionella jeoni* [4]. Despite being a diverse group, approximately half of the described species has been associated with respiratory human diseases [1,5]. They are often found in drinking water distribution systems such as showers, baths, heating systems or in re-cooling plants, cooling towers, air conditioning units and decorative water items, where aerosolization, dissemination and transmission can occur [6,7]. Due to their capability to form biofilms and replicate within eukaryotic cells like amoeba or alveolar macrophages, their survival rate to disinfectants is high and makes *Legionella* a large public health threat [6,8].

In Germany, the presence of *Legionella* in water installations is constantly monitored by the public health authorities responsible for the region administration, following the guidelines from the DIN EN ISO 19458:2006-12 (Water quality – Sampling for microbiological analysis, ISO 19458:2006). In this study, we present two *Legionella* strains isolated from a re-cooling plant and from a drinking water installation, which, based on genomic, phylogenetic and phenotypic analyses, represent two novel species within the genus *Legionella*.

## Methods

### Isolation and cultivation

Water samples were collected from a re-cooling plant in Friolzheim and from a drinking water installation in Lahr, Germany, in October 2022 and September 2024, respectively. The samples were collected according to DIN EN ISO 19458:2006; Category b) by the Local Health Office from each region and analysed by the State Health Office of Baden-Württemberg. The isolation of *Legionella* species was performed by a standard culture technique on glycine–vancomycin–polymyxin B–cycloheximide (GVPC) agar medium following DIN EN ISO 11731:2019-03. Fifty millilitres of the water sample was filtered through a membrane filter (0.45 µm). After filtration, the membrane filters were coated with an acid buffer solution (0.2 M HCl/KCl, pH 2.0–2.2) and left to act for 5 min. The buffer was aspirated and rinsed with a Ringer’s nutrient solution (1:10). The filters were placed on a GVPC agar plate and incubated, and growth was monitored after 5, 7 and 10 days of incubation. Presumptive *Legionella* colonies were sub-cultured on buffered charcoal yeast extract agar (BCYE) with cysteine and in tryptone soya agar with 5% sheep blood agar medium (Thermo Fisher Scientific Diagnostic). Cultivation and sub-cultivation were performed aerobically at 36±1 °C under an atmosphere of 95% relative humidity. Isolates were preserved at −80±2 °C in Cryobank tubes with Cryobeads following the manufacturers’ instructions.

### Identification and genome sequencing

The sub-cultures that did not grow in blood agar medium, considered suspicious to be *Legionella* isolates, were tested using the Oxoid Legionella Latex Test (Part No. DR0800M, Thermo Fisher Scientific Diagnostic), a qualitative latex slide agglutination assay used for the identification of some serogroups of *Legionella* species: *Legionella longbeachae* serogroup 1 and 2, *Legionella bozemanii* serogroup 1 and 2, *Legionella dumoffii*, *Legionella gormanii*, *Legionella jordanis*, *Legionella micdadei* and *Legionella anisa. Legionella*-suspected isolates were identified with the matrix-assisted laser desorption ionization time-of-flight MS technique with the VITEK MS PRIME system (bioMerieux) following the manufacturer’s instruction.

DNA extraction was performed following a protocol for preparation of genomic DNA using mechanical lysis [9]. Briefly, cells were grown and resuspended in PBS to a density of 1.0 (A600nm). The cell pellet was suspended on 0.15 ml of PBS buffer and incubated at 99 °C for 5 min, followed by a mechanical lysis using acid-washed glass beads (425–600 µm) and a cell disruptor (frequency: 30 Hz) for 10 min. The tubes were then centrifuged at 12,000 ***g*** for 5 min. The supernatant was recovered and diluted in 100 µl of nuclease-free water and quantified using fluorometric quantification (Qubit, Thermo Fisher Scientific).

*Legionella*-suspected isolates were analysed by amplifying the 16S rRNA gene sequences using the universal primers 27F and 1492R [10]. Amplicons were Sanger sequenced and aligned with sequences from *Legionella*-related species at the non-redundant GenBank 16S ribosomal RNA database from the National Center for Biotechnology Information (NCBI). Phylogenetic trees were generated based on maximum-likelihood using Unipro UGENE software (version 53.0) and iTOL (version 6.5.8) [11,12]. Whole-genome sequencing was performed using the library preparation of MiSeq DNA library prep kit from Illumina and sequenced using a 150 bp paired-end run on an Illumina MiSeq platform (Illumina). Reads were quality-controlled, decontaminated from sequencing artefacts or adapters and merged using BBtools (version 37.62) [13]. Assembly was performed using SPAdes (version 3.15.0) [14] under a high-coverage isolate tag for filtered and merged reads. The quality of the assembled genome was assessed using QUAST (version 5.3.0) [15], and completeness and contamination levels were further verified using CheckM2 (version 1.2.2) (https://github.com/Ecogenomics/CheckM). The genome assemblies and raw files were submitted to the European Nucleotide Archive (ENA) database under the BioProject number PRJEB90345. Genome annotation and gene prediction was done with Prokka (version 1.14.5) [16]. Secondary metabolite biosynthetic gene clusters were analysed with antiSMASH 8.0 database [17].

### Physiology and chemotaxonomy

Strains were analysed under an automated system for characterization of biochemical features using Vitek 2 Compact System version 9.04.3 (bioMerieux). The test panels contained 47 colorimetric tests that included the detection of aminopeptidases, decarboxylase tests (for ornithine and arginine) and additional miscellaneous tests (urease, beta-glucuronidase, Ala-Phe-Pro acrylamidase or Pyrrolidonyl Arylamidase). The card was sealed and inserted into the VITEK 2 reader-incubator module (35.5 °C incubation temperature) and subjected to a kinetic fluorescence measurement every 15 min. The results were interpreted by the ID-BCL database.

Cellular fatty acid (CFA) determination was performed by the DSMZ services Leibniz-Institut DSMZ–Deutsche Sammlung von Mikroorganismen und Zellkulturen GmbH (Braunschweig, Germany). A biomass pellet of the strains WA2022007384^T^ and WA2024007413^T^ was collected from BYCE agar plates of 72 h incubation, frozen and sent for analysis to the DSMZ services, following the standard protocol recommended for the MIDI Microbial Identification System (MIDI Inc., version 6.1, Newark, DE). The composition of CFAs was identified using the Sherlock microbial identification system (TSBA40 library; MIDI Inc.).

### Taxonomy analysis

Genomes were taxonomically classified with GTDB-Tk (version 2.1.0) [18,19] with the database GTDB (R220) [20]. Genome contigs were submitted to the Type (Strain) Genome Server (TYGS), for a whole genome-based taxonomic analysis confirmation [21] using the fast distance-based phylogeny inference programme FastME 2.0 [22]. Since the isolates were not assigned to a reliable taxonomic identity, they were further analysed using genomic comparison to all members of the genus *Legionella*. Genomes of all species of *Legionella* were collected using the NCBI genome browser (https://www.ncbi.nlm.nih.gov/datasets/genome/) considering type strains according to the LPSN. Phylogenetic trees were constructed based on 164 concatenated core genes using the concatenated-gene-alignment-fasta tool from Anvi’o version 8.1 [23], inferred by maximum-likelihood using FastTree version 2.1.11 [24]. The generated trees were visualized with iTOL (version 6.5.8) [12] and annotated with the tool table2itol in R studio version 3.6.1 (https://github.com/mgoeker/table2itol). The identity parameters for taxonomic delineation, digital DNA–DNA hybridization (dDDH) and average nucleotide identity (ANI), were calculated using the Genome-to-Genome Distance Calculator (GGDC version 3.0) (https://ggdc.dsmz.de/) from the DSMZ [25] and the software FastANI version 1.33 (https://github.com/ParBLiSS/FastANI), respectively. Accession numbers of the reference genomes used in the analysis are provided in Table S1 (available in the online Supplementary Material).

## Results and discussion

**Fig. 1.**
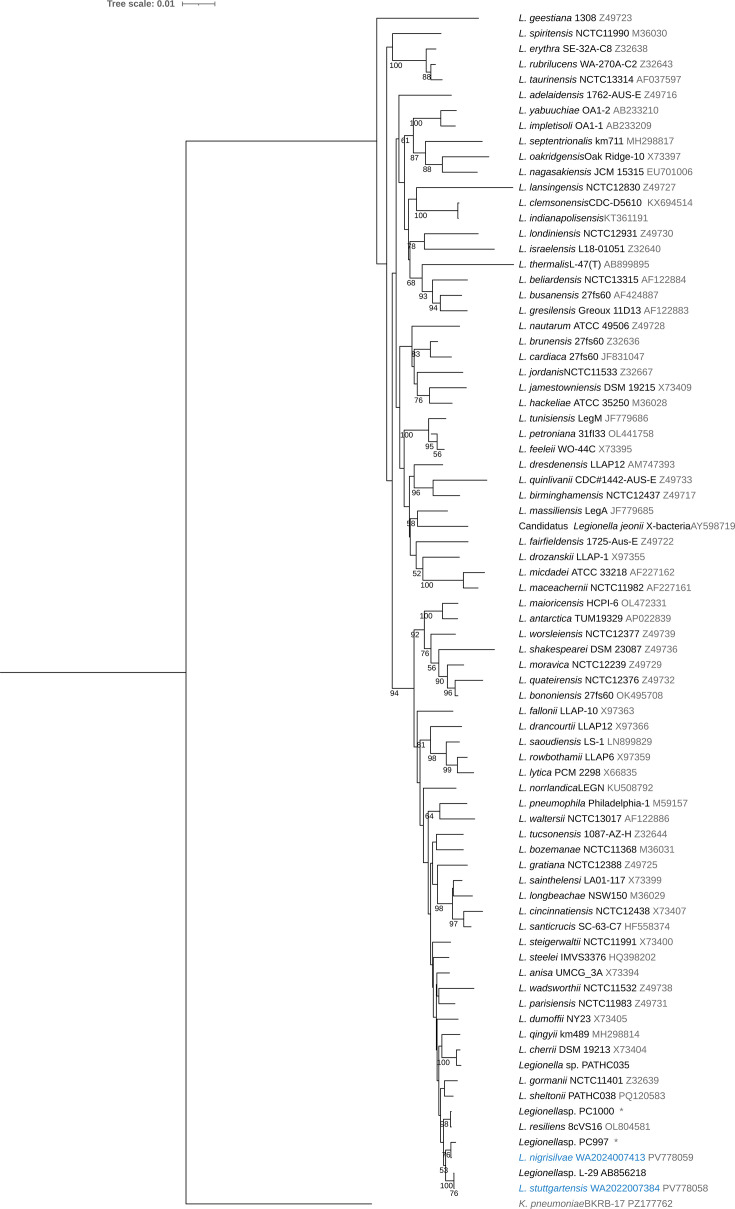
Maximum-likelihood tree showing the relationship between strains WA2022007384^T^ and WA2024007413^T^ to species of *Legionella* based on a 16S rRNA gene alignment (1,400 bp) using *Klebsiella pneumoniae* BK-RB-17 as an outgroup, created with Unipro UGENE software. Strains of novel species are highlighted in blue. Branch lengths correspond to sequence differences indicated by the scale bar. GenBank accession numbers and strain names are given for each leaf. *The 16S sequence of the strain PC997 and PC1000 were retrieved from the genome sequence using ContEst16S [37].

The strains described in this work were isolated within a routine detection of *Legionella* sp. from a re-cooling plant and from a drinking water installation, performed by the Local Health Office of Friolzheim and Lahr/Schwarzwald, Germany, and analysed by the Health Office of the state of Baden-Württemberg. The strain WA2022007384 was isolated from a water sample taken in October 2022, and the strain WA2024007413 was obtained from a water sample taken in September 2024. The routine identification using MS (Vitek MS Prime, bioMerieux) did not provide an identity match as for other common species of *Legionella*, *L. pneumophila* or *L. anisa*, and depicted a similarity match of 96% to *Pasteurella cannis*. However, the differential test of growth in blood agar resulted negative for both strains. The 16S rRNA sequences of the strain WA2022007384 showed a 100% identity to *Legionella* sp. L-29 and 99.41% to *Legionella* sp. PC997, two strains not assigned to a species. In contrast, the sequence of WA2024007413 exhibited 99.92% sequence identity to *Legionella* sp. PC997 and 99.2% to *Legionella resiliens* 8cVS16 (Fig. 1).

### Genomic features

**Fig. 2.**
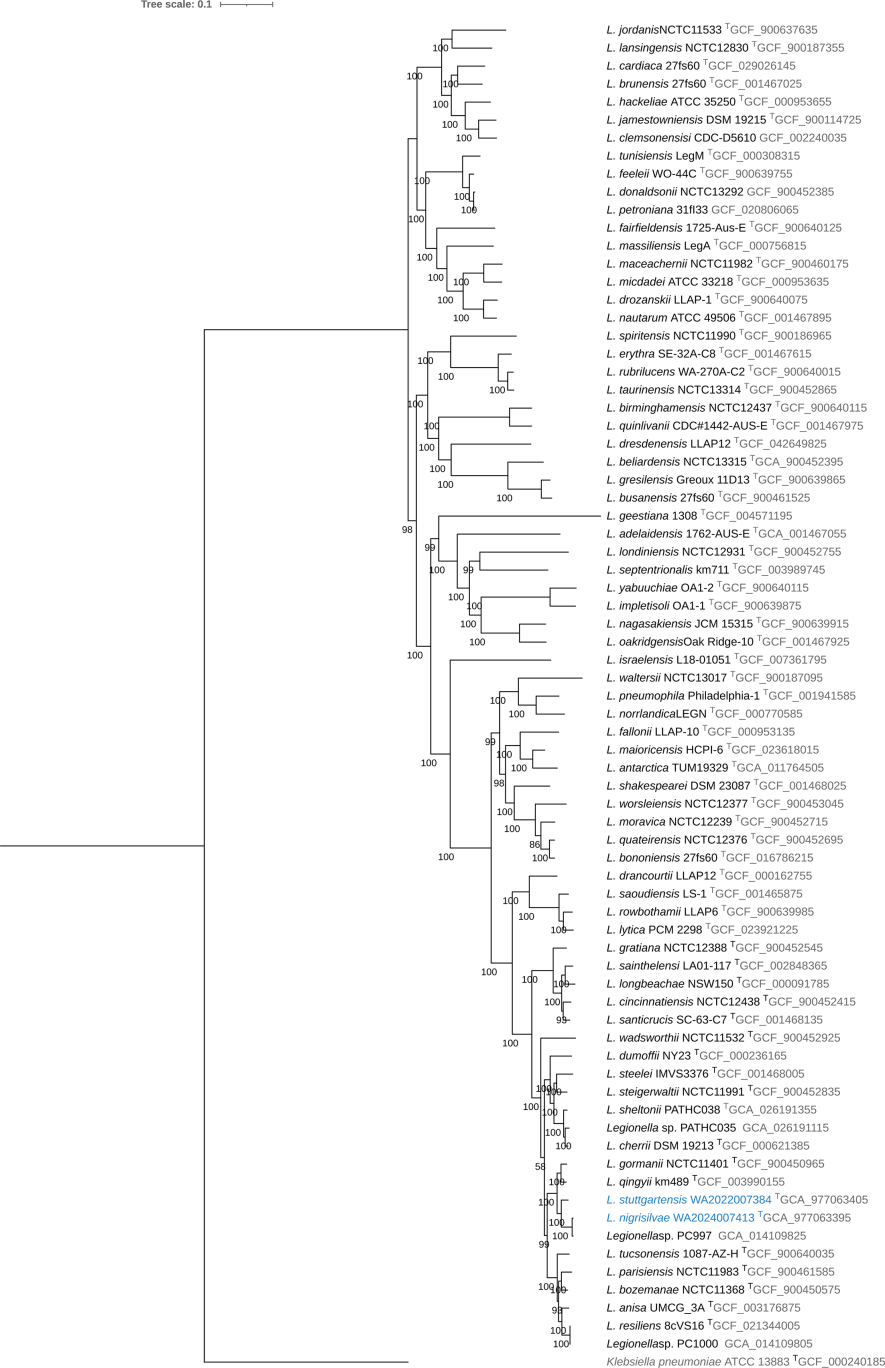
Maximum-likelihood phylogenomic tree of Legionella based on 164 concatenated core genes, with *L. stuttgartensis* and *L. nigrisilvae* as novel species, rooted with Klebsiella pneumoniae ATCC 13883T. Strains of novel species WA2022007384^T^ and WA2024007413^T^ and are highlighted in blue. Bootstrap values (1,000 replicates) are depicted above branches, and branch lengths correspond to sequence differences indicated by the scale bar.

The genome of WA2022007384 was assembled in 59 contigs and encompassed 4,334,595 bp with a 37.92 GC mol%. The number of coding DNA sequences (CDS) was 3,807, including 3 rRNA and 42 tRNA genes. The genome metrics of both strains lay within the ranges of the genus *Legionella* and approach similar ranges within the neighbour strains according to the phylogram (Fig. 2). The genome assembly of the strain WA2024007413 included 26 contigs and 3,881,291 bp. The GC mol% was 37.91 and the CDS of 3434, including 3 rRNA and 44 tRNA genes. The N50 of both assemblies was 465 and 817, respectively.

The genome of both species encoded the gene resistance cluster blaFEZ-1 coding for a metallo-*β*-lactamase operon, already reported in other species of *Legionella*. It might confer resistance against cephalosporin, penicillin and carbapenem when expressed [26]. Additionally, the strain WA2024007413 encoded two more antibiotic resistance clusters: an aminoglycoside phosphotransferase APH(9)-Ia and the *β*-lactamase OXA-29 that will confer resistance to aminoglycosides and penicillin-like antibiotics, respectively. All resistance gene clusters identified among the two species have been described in *L. gormanii* [27] and *L. pneumophila* [28].

### Phylogenomic analysis

**Fig. 3.**
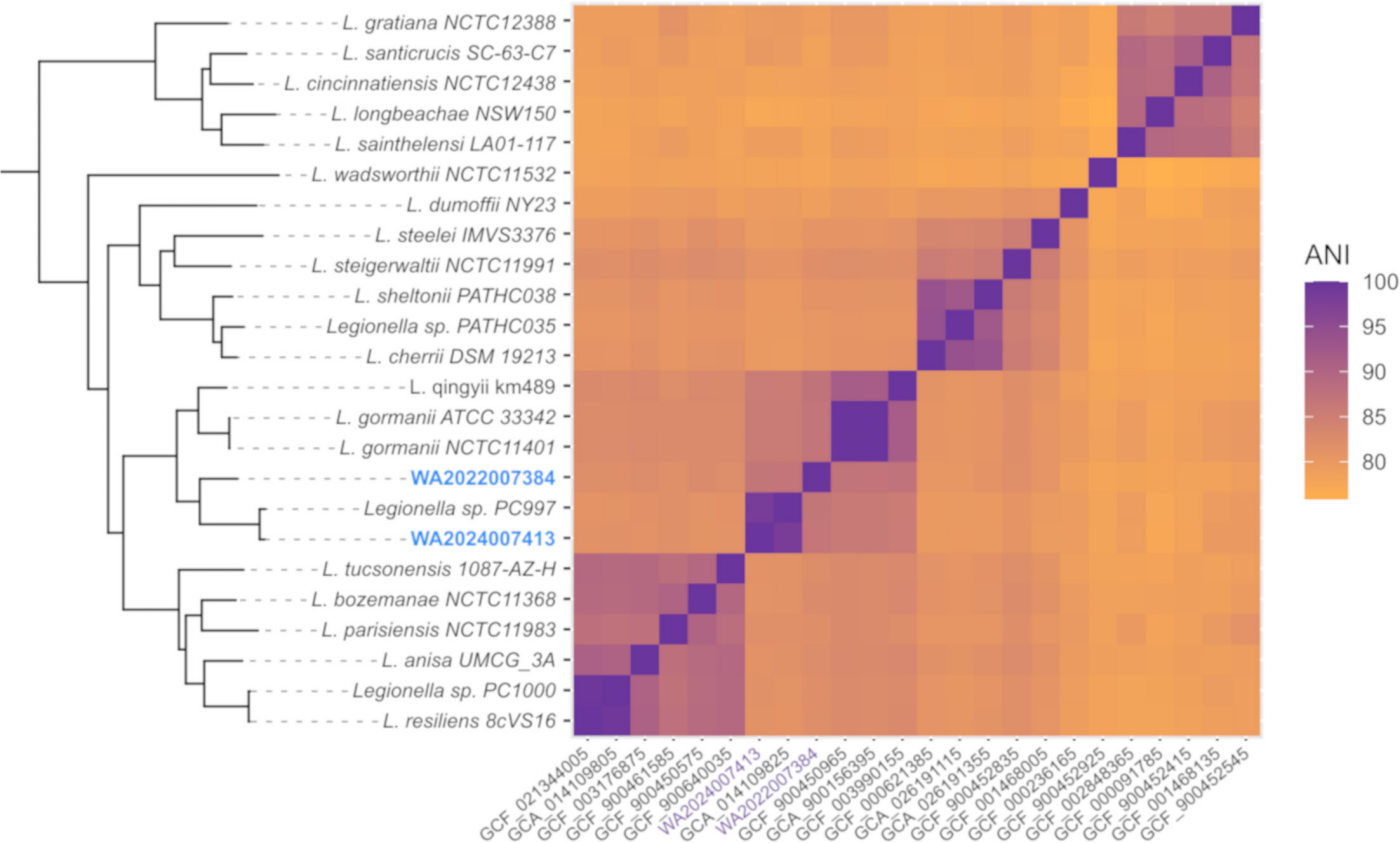
Correlation matrix of average amino acid identity scores (ANI) calculated among reference species closely related to the strains WA2022007384^T^ and WA2024007413^T^. ANI percentage corresponds to colour indicated by the scale bar on the right side.

The phylogenetic analysis based on 16S rRNA gene (Fig. 1) and the phylogenomic analysis based on 164 concatenated core genes (Figs 2 and S1) confirmed that the strains WA2022007384 and WA2024007413 belong to a single clade neighbouring species of *Legionella qingyii* [29] and *L. gormanii* [30]. The strain WA2022007384 exhibited 87.41% ANI to *L. qingyii* km489^T^ and 86.97% to *L. gormanii* NCTC11401^T^, while the strain WA2024007413 presented 98.28% ANI to *Legionella* sp. PC997, 85.84% to *L. gormanii* NCTC11401^T^ and 85.61% to *L. qingyii* km489^T^. The high identity ANI percentage to the strain PC997 of *Legionella* sp. [31] lies within the intra-species threshold of >95% [32]. The genome of the strain PC997 has been reported as an environmental isolate of *Legionella*. However, it has not been validly described as a novel species [31]. The high ANI percentage of the strain PC997 to our isolate WA2024007413 and the topology observed in the phylogenetic tree suggest that both isolates belong to the same species: *Legionella nigrisilvae* sp. nov. Despite the similarities among the strains WA2022007384 and WA2024007413, the ANI percentage stayed within the inter-species threshold with 86.81%, confirming the description of two different species of *Legionella* (Fig. 3, Table S2).

The *in silico* GGDC estimated the strain WA2022007384 to have a 51.70% dDDH similarity to the genome of *L. gormanii* NCTC11401^T^, 48.00% to *Legionella qingyii* km489^T^ and 55.30% to the strain *Legionella* sp. PC997, with a distance of 0.3220, 0.2985 and 0.2699, respectively. The strain WA2024007413 presented a dDDH similarity of 51.70% to the genome of *L. gormanii* NCTC11401^T^, 46.50% to *Legionella qingyii* km489^T^ and 90.80% to the strain Legionella sp. PC997, with a respective distance of 0.2943, 0.3338 and 0.0764. A distance bigger than 0.258 was considered as a threshold for species delineation [25].

### Physiology, cell morphology and chemotaxonomy

**Fig. 4.**
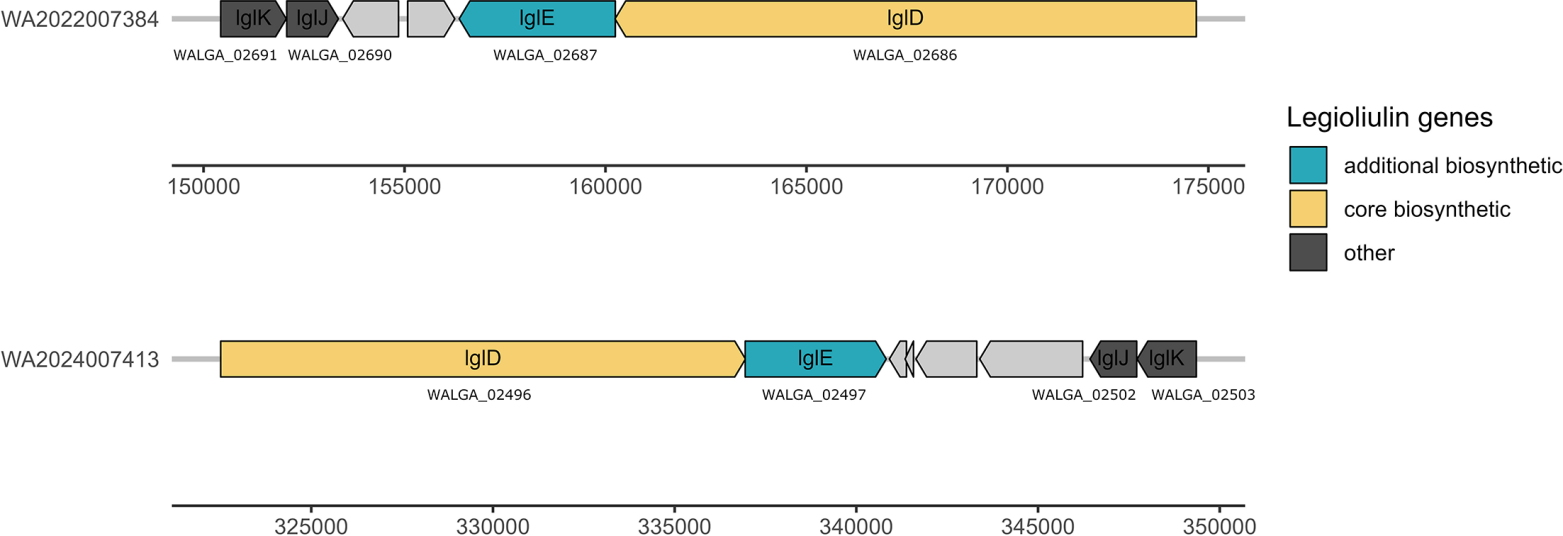
Gene cluster distribution of legioliulin genes, potentially responsible for the blue fluorescence observed under UV light at 365 nm, encoded in *Legionella stuttgartensis* WA2022007384^T^ and *L. nigrisilvae* WA2024007413^T^. The annotation files are available under the accessions GCA_977063405.2 and GCA_977063395.2.

The subculture of both strains was performed on BCYE-Cys agar plates with activated charcoal. Bacterial colonies were visible within 5 to 10 days as white convex colonies of defined entire edges of 2 to 5 mm in diameter of moist shiny appearance and fluorescent under UV light at 365 nm (Fig. S2). Both strains resulted negative to the Gram stain test and present thin small rods of ~0.3 to 0.5 µm wide and 5 to 10 µm long. The strain WA2024007413 presented a microscopic morphology of rods arranged in long chains, while the strain WA2022007384 showed an aleatory rod distribution. Both strains produced colonies that showed a light blue colour under UV light at 365 nm and blue fluorescence in darkness under UV light at 365 nm (Fig. S2). Fluorescent emission is common in some species of environmental *Legionella* and has been proposed as a confirmation method for detecting *Legionella* colonies isolated from water samples [33]. The genes responsible for the colony’s fluorescence *lgl* (legioliulin) were annotated within the secondary metabolism annotation, these biosynthetic clusters might be involved in the biosynthesis of a natural fluorophore [34]. Legioliulin genes were encoded within both genomes (Fig. 4) and might be responsible for the production of fluorescence under UV light at 365 nm.

The annotation files are available under the accessions GCA_977063405.2 and GCA_977063395.2. The agglutination test resulted negative for both strains, and none of the strains grew in blood agar as a differential test. The panel of biochemical parameters analysed by Vitek 2 (bioMerieux) resulted in a negative expression of aminopeptidases, carbohydrate utilization and decarboxylase tests. However, both strains tested positive for the expression of phosphatase activity (Table S3).

Major CFAs detected were branch-chain acids. The strain WA2022007384^T^ presented 34.2% of saturated anteiso-C_15 : 0_, 18.8% of iso-C_16 : 0_, 12% of C_16:1_ *ω*7c/C_16:1_ *ω*6c, 6.7% of anteiso-C_17:0_ and 6.3% of iso-C_14 : 0_. The strain WA2024007413^T^ showed 30.8% of saturated anteiso-C_15 : 0_, 26.2% of iso-C_16:0_, 11.5% of anteiso-C_17 :0_ and 8.3% of C_16:1_ *ω*7c/C_16:1_ *ω*6c. The fatty acid profile showed a very similar spectrum to that of the genus *Legionella*, with dominating a15:0, i16:0 and a17:0 [35]. The major fatty acids of WA2022007384^T^ and WA2024007413^T^ have a similar pattern percentage of fatty acids to species of *L. gormanii*, *L. dumoffii and L. qingyii* [36], with a distinctive difference of presenting a higher percentage of C_16:1_ *ω*7c/C_16:1_ *ω*6c than other *Legionella* species. Table 1 shows the CFA profile content of strains that neighbour the strains WA2022007384^T^ and WA2024007413^T^ within the same clade. Additional fatty acid profile of other *Legionella* species (*L. dumoffii* ATCC 33279^T^, *L. anisa* ATCC 35292^T^, *Legionella parisiensis* ATCC 35299^T^, *Legionella bozemanae* ATCC 33217^T^, *Legionella tucsonensis* ATCC 49180^T^ and *L. resiliens* 8cVS16^T^) is shown in Table S4.

**Table 1.** CFA compositions of strains WA2022007384^T^, WA2024007413^T^, *L. qingyii* km488^T^ and *L. gormanii* ATCC 33297^T^

Fatty acid	WA2022007384^T^ DSM 120481	WA2024007413^T^ DSM 120480	*L. qingyii* km488^T^	*L. gormanii* ATCC 33297**^T^**
**Saturated straight-chain**				
C_14 : 0_	0.4		2.0	–
C_15 : 0_	3.9	0.6	3.3	2.3
C_16 : 0_	3.5	1.6	8.3	9.6
C_17 : 0_	1.8	1.0	4.8	3.6
C_18 : 0_	0.6	0.5	1.9	1.5
C_19 :0_	0.4	0.4	–	–
C_20 : 0_	0.3	0.7	–	0.7
**Saturated branched-chain**				
Anteiso-C_11 : 0_	0.2			
Anteiso-C_13 : 0_	0.2			
Iso-C_14 : 0_	6.3	4.8	2.9	4.3
Iso-C_15 : 0_	0.9	0.6	–	–
Anteiso-C_15 : 0_	34.2	30.8	25.9	22.1
Iso-C_16 : 0_	18.8	26.2	14.5	19.4
Iso-C_17 : 0_	0.5	0.5	–	0.7
Anteiso-C_17 : 0_	6.7	11.5	13.8	9.9
Iso-C_18 : 0_	0.2	0.8		
Anteiso-C_19 : 0_		0.3		
Iso-C_20 : 0_		0.2		
**Branched-chain hydroxy**				
C_13 :0_ iso 3OH	0.2			
C_14:0_ iso 3OH				
C_16:0_ iso 3OH	0.2	0.2		
**Unsaturated/hydroxy**				
C_14 : 1_ *ω*5c	0.2	0.2		
C_15 : 1_ *ω*4c	1.0			
C_15 : 1_ *ω*6c	3.0	1.3	1.8	2.1
C_16 : 1_ *ω*5c	1.5	0.8	–	–
C_16 : 1_ *ω7*c				
C_16 : 1_ *ω*11c				
C_17 : 0_ cyclo	1.1	7.4	4.8	9.6
C_18 : 1_ *ω*7c			–	–
**Summed features**				
2 (C_16 : 1_ iso I/C_14:0_ 3OH)	0.3	0.2		
(C_16:1_* ω*7c/C_16:1_* ω*6c)	12	8.3		
(C_16:1_* ω*6c/C_16:1_* ω*7c)	0.3			
(C_18:1_* ω*7c/C_18:1_* ω*6c)	0.4			

(-) Not available Data is marked with a middle hyphen.

## Protologue

### Description of *Legionella stuttgartensis* sp. nov.

*Legionella stuttgartensis* (N.L. fem. adj. *stuttgartensis*, pertaining to Stuttgart).

This species was isolated in Stuttgart by the Public Health department of the state Baden-Württemberg from a re-cooling tower in the municipality of Friolzheim. *Legionella stuttgartensis* is a Gram-negative, oxidase- and catalase-positive aerobic rod-shaped bacterium. It produces white colonies at 35–37 °C on BCYE medium with cysteine. The type strain genome of *Legionella stuttgartensis* WA2022007384^T^ (=DSM 120481^T^=NCCB 101094^T^) has a G+C content of 37.92 mol% and encodes 3,807 genes. Major CFAs are anteiso-C_15 : 0_, iso-C_16 : 0_, C_16:1_ *ω*7c/C_16:1_ *ω*6c, anteiso-C_17 : 0_ and iso-C_14 : 0_.

The 16S rRNA gene GenBank accession number of strain WA2022007384^T^ is PV778058. The GenBank accession number for the whole-genome shotgun project is GCA977063405.

### Description of *Legionella nigrisilvae* sp. nov.

*Legionella nigrisilvae* (L. masc. adj. *niger*, black; L. fem. n. *silva*, forest; N.L. gen. n. *nigrisilvae*, of the Black Forest).

Gram-negative, oxidase- and catalase-positive aerobic rod-shaped bacterium species isolated in Stuttgart by the Public Health department of the state Baden-Württemberg from a drinking water installation taken in the district of Lahr/Schwarzwald (Black Forest) in Germany. *L. nigrisilvae* is a Gram-negative stain, oxidase and catalase-positive aerobic rod-shaped bacterium. It produces white colonies after 5 to 10 days of incubation at 35–37 °C on BCYE medium with cysteine. The type strain genome of *L. nigrisilvae* WA2024007413^T^ (=DSM 120480^T^=NCCB 101095^T^) has a G+C content of 37.91 mol% and encodes 3,434 genes. Major CFAs are anteiso-C_15 : 0_, iso-C_16:0_, anteiso-C_17 : 0_ and C_16:1_ *ω*7c/C_16:1_ *ω*6c.

The 16S rRNA gene GenBank accession number of strain WA2024007413^T^ is PV778059. The GenBank accession number for the whole-genome shotgun project is GCA 977063395.

## Supplementary material

10.1099/ijsem.0.007187 Supplementary Material 1.

10.1099/ijsem.0.007187 Supplementary Material 2.

## Abbreviations

ANI: average nucleotide identity
ANI: average nucleotide identity
BCYE: buffered charcoal yeast extract agar
CFA: cellular fatty acid
Cys-: without l-cysteine
Cys+: with l-cysteine
dDDH: digital DNA–DNA hybridization
GGDC: Genome-to-Genome Distance Calculator
GVPC: glycine–vancomycin–polymyxin B–cycloheximide agar medium
LPSN: List of Prokaryotic Names with Standing in Nomenclature
NCBI: National Center for Biotechnology Information
