# Supplementary material for: Legionella stuttgartensis sp. nov. and Legionella nigrisilvae sp. nov., two new species isolated from a re-cooling plant and from a drinking water house installation in southern Germany
Source: Int J Syst Evol Microbiol. 2026 Jun 2;76(6):007187. doi: 10.1099/ijsem.0.007187 (PMC13229132; doi:10.1099/ijsem.0.007187)
Supplement: Supplementary Material 1. [file ijsem-76-07187-s001.pdf]

Supplementary Material

Legionella stuttgartensis sp. nov., and Legionella nigrisilvae sp. nov., two new species isolated from a recooling plant and from a drinking water house installation in southern Germany

Bibiana Rios-Galicia[1*], Patryk Krauze[1], Caroline Hengerer[1], Konstantin Licht[1], Stefan Brockmann[1], Jens Fleischer[1*]

[1] Ministerium für Soziales, Gesundheit und Integration, Landesgesundheitsamt, Baden-Württemberg Nordbahnhofstr. 135, 70191 Stuttgart, Germany

*Correspondence: Bibiana Rios-Galicia, bibiana.riosgalicia@sm.bwl.de

*Correspondence: Jens Fleischer, Jens.Fleischer@sm.bwl.de

The supplementary material encompass:

- A list of three supplementary tables available in format .xlsx (Supplementary Tables).

-Two supplementary figures with description (Supplementary figures S1 and S2).

Supplementary Tables

Table S1. Reference genomes used for the phylogenetic analysis of *Legionella stuttgartensis* sp. nov. and *Legionella nigrisilvae* sp. nov.

Table S2. Animo acid Nucleotide Index and 16S identity percentage of the strains WA2022007384^T and WA2024007413^T against species of *Legionella.*

Table S3. Phenotypic characterization of the strains WA2022007384^T and WA2024007413 ^{T} using the system VITEK® 2.

Table S4. Cellular fatty acid compositions of strains WA2022007384^T, WA2024007413^T, *L.qingyii* km488^T, *L. gormanii* ATCC 33297^T, *L. dumoffii* ATCC 33279^T, *L. anisa* ATCC 35292^T, *L. parisiensis* ATCC 35299^T, *L. bozemanae* ATCC 33217^T, *L. tucsonensis* ATCC 49180^T and *L. resiliens* 8cVS16^T.

Supplementary Figures

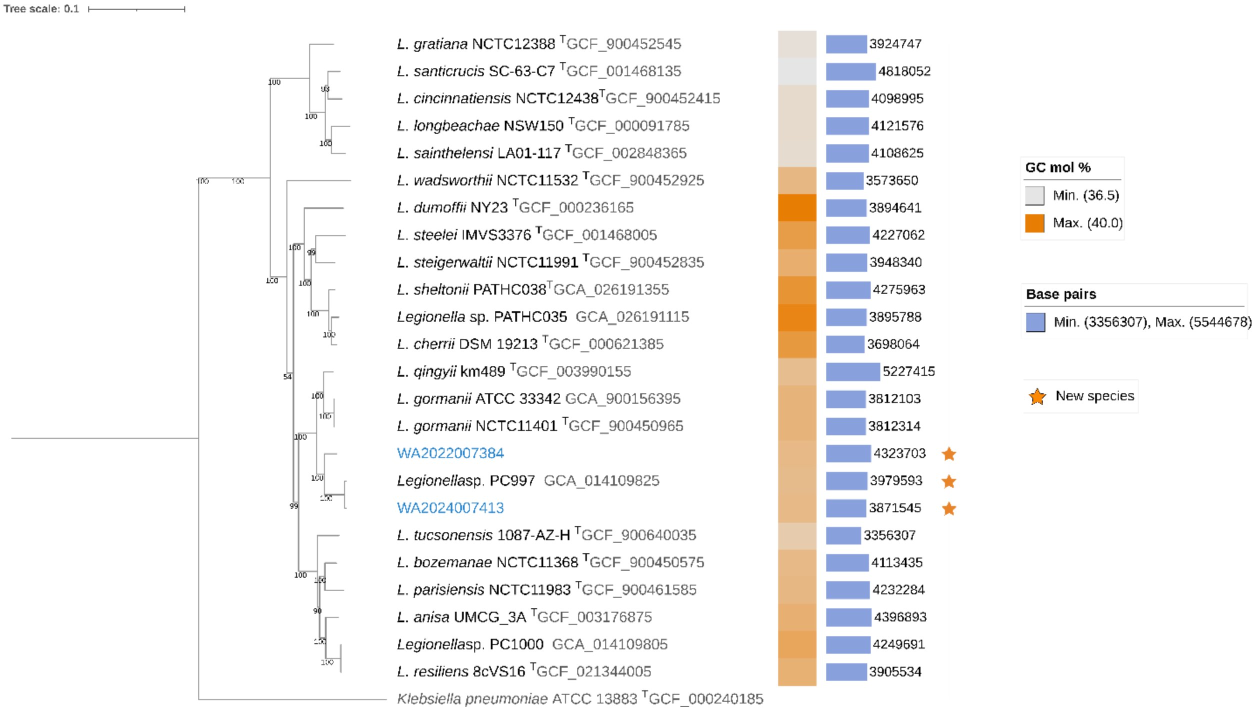

Figure S1. Annotated phylogenomic tree of type species of *Legionella* based on 164 concatenated core genes obtained from close related genomes. Strains of novel species WA2022007384^{T} and WA2024007413^{T} are highlighted in blue. The tree was inferred by the maximum-likelihood method and visualized with iTol using *Klebsiella pneumoniae* ATCC 13883T as an outgroup. Bootstrap values (1000 replicates) are depicted above branches and branch lengths correspond to sequence differences indicated by the scale bar.

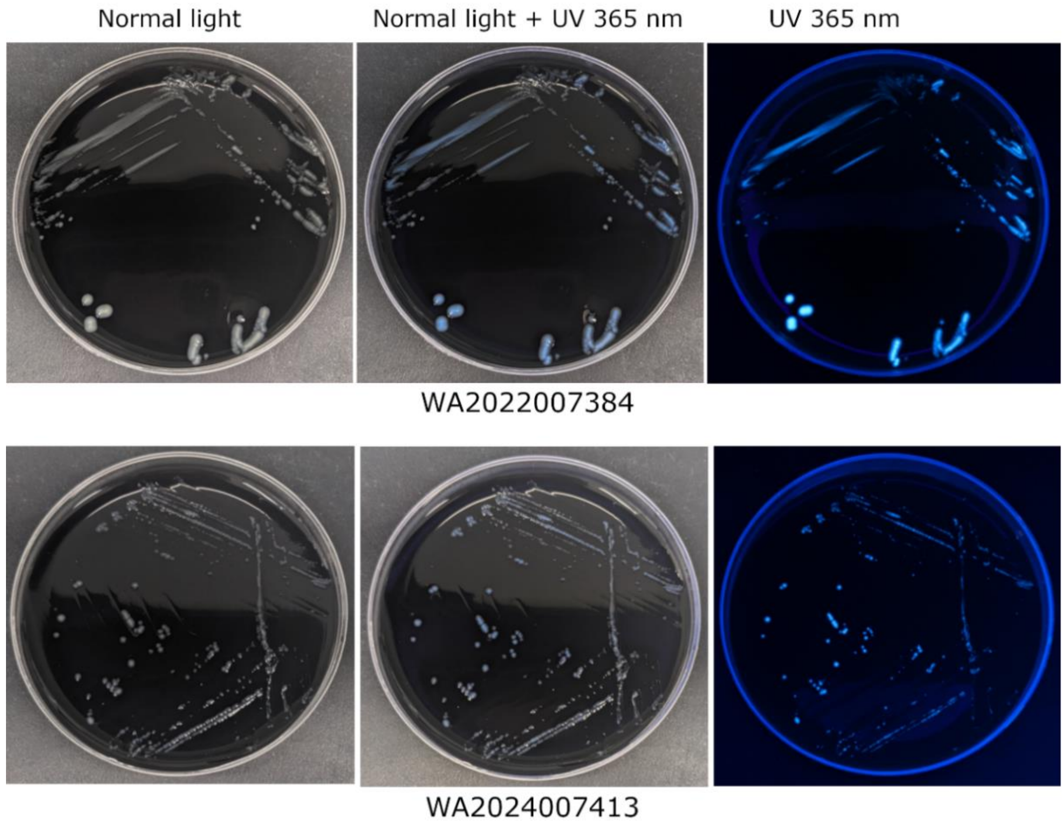

Figure S2. Growth morphology of *Legionella stuttgartensis* WA2022007384^T and *Legionella nigrisilvae* WA2024007413^T on buffered charcoal yeast extract (BCYE) agar: From left to right, the plates depict both strain's growth observed under normal light, UV light at 365 nm, and UV light at 365 nm in darkness. The plates show colonies after a 7 days incubation at 37 °C.
